# Transcriptomic Approach in Understanding Fabry Nephropathy: A Review of the Literature and Proof-of-Concept

**DOI:** 10.3390/genes16050601

**Published:** 2025-05-19

**Authors:** Nika Breznik, Tina Levstek, Bojan Vujkovac, Andreja Cokan Vujkovac, Katarina Trebušak Podkrajšek

**Affiliations:** 1 Institute of Biochemistry and Molecular Genetics, Faculty of Medicine, University of Ljubljana, 1000 Ljubljana, Slovenia; 2 Clinical Institute for Special Laboratory Diagnostics, University Children’s Hospital, University Medical Centre Ljubljana, 1000 Ljubljana, Slovenia; 3 Centre for Fabry Disease, General Hospital Slovenj Gradec, 2380 Slovenj Gradec, Slovenia

**Keywords:** Fabry nephropathy, RNA sequencing, transcriptomic, Fabry disease, lysosomal storage disease

## Abstract

Background/Objectives: Fabry nephropathy (FN) is a progressive complication of Fabry disease that significantly affects patient outcomes. However, the molecular mechanisms underlying FN are not yet fully understood. Recent advances in transcriptomics have opened new perspectives for the identification of early changes in gene expression associated with the development and progression of the disease. Methods: This study includes a systematic review of transcriptomic findings in chronic kidney disease, with a particular focus on FN, and presents a proof-of-concept RNA sequencing analysis of peripheral blood samples from six Fabry patients with progressive nephropathy and six age- and sex-matched control subjects. Results: The analysis identified 41 differentially expressed genes (DEGs), all of which were upregulated in Fabry patients. Enrichment analysis revealed significant involvement in immune-related pathways, including neutrophil degranulation, interferon, and cytokine signaling. Cell type enrichment revealed that neutrophils and other immune cells are key players in this process. Conclusions: These results suggest that immune and inflammatory mechanisms play a central role in the pathogenesis of FN. The identified DEGs are involved in pro-fibrotic signaling and immune system activation and shed light on possible mechanisms underlying fibrosis, podocyte injury, and kidney damage. This study contributes to a deeper understanding of FN and may facilitate in the identification of early biomarkers for diagnosis and disease monitoring.

## 1. Introduction

Fabry nephropathy (FN) is one of the most important complications of Fabry disease (FD), although its mechanism is still relatively poorly understood. In untreated patients, kidney involvement typically manifests in the second or third decade of life and initially presents with albuminuria and proteinuria [1]. However, histological studies in pediatric patients with FD have shown kidney damage before the onset of symptoms [2], suggesting that FN begins much earlier than previously thought. Untreated Fabry patients often develop chronic kidney disease (CKD), which may progress to end-stage kidney disease, before the age of 50 [3]. Before the introduction of kidney replacement therapy, end-stage kidney disease was the leading cause of premature death in Fabry patients [4]. Although the introduction of enzyme replacement therapy (ERT) showed that it can stabilize kidney function, it cannot prevent further deterioration in patients with advanced nephropathy [5]. Importantly, FN significantly affects patient survival as it increases the risk of dysfunction in other organ systems [1]. The clinical manifestations of FN show considerable variability between patients, even between those from the same family carrying the same pathological variant in the *GLA* gene [6]. This suggests that additional, yet unidentified, modifying factors contribute to the development and progression of FN.

Recent advances in molecular research have provided a deeper understanding of the pathophysiological mechanisms underlying FD. In this context, transcriptomics has emerged as a powerful tool to study gene expression profiles that have been shown to be influenced by genetic, physiological, and environmental factors. Transcriptome analysis facilitates the identification of differentially expressed genes (DEGs) and dysregulated biological pathways involved in the development and progression of FN. This approach enables the identification of early molecular alterations that precede clinical and histological manifestations, facilitating the discovery of novel biomarkers and potential therapeutic targets.

This article aims to provide a comprehensive review of the current transcriptomic studies in patients with CKD, with a focus on FN. It also presents the results of a proof-of-concept study employing RNA sequencing of peripheral blood samples to investigate the transcriptomic factors that contribute to the development of FN.

### 1.1. Transcriptomic Studies in Chronic Kidney Disease

In CKD, altered gene expressions have been observed even before histological changes [7]. Kidney biopsies from patients with progressive CKD revealed the upregulation of inflammatory pathways involving genes such as *CCL19*, *CXCL1*, *IFNAR2*, *NCK2*, *PTK2B*, *PTPRC*, *RASGRP1,* and *TNFRSF25*, as well as an increased expression of transcripts involved in apoptosis, including *MAP3K14*, *TNFRSF10B*/*TRAIL-R2*, *TRADD*, and *TRAF2* [8]. Transcriptomic profiling of blood samples from CKD patients has shown that uremia profoundly alters the gene expression related to various cellular processes, namely the downregulation of pathways, such as mRNA and protein transport, chaperone function, the unfolded protein response, tumorigenesis, cytoskeletal remodeling, endocytosis, T-cell and immune signaling, and the upregulation of pathways involved in insulin-like growth factor activity, neuroactive receptor interaction, the complement system, lipid metabolism, and the ubiquitin–proteasome system [9].

Transcriptomic studies on kidney biopsy samples in diabetic kidney disease (DKD) have revealed 84 upregulated and 49 downregulated DEGs, with the enrichment of pathways related to extracellular matrix interactions, focal and cell adhesion, pathogen response among the upregulated genes, and metabolic and mineral absorption processes among the downregulated ones, while hub genes predominantly localized to immune cells, highlight the key role of inflammatory infiltration in DKD pathogenesis [10]. Additional studies further support this by identifying innate immune activation, particularly the upregulation of FcER1 signaling in both blood and kidney tissue, as a central mechanism in DKD progression and a potential target for therapeutic intervention [11].

### 1.2. Transcriptomic Studies in Fabry Disease

While the metabolic origin and rate of progression of FN are similar to those of DKD, transcriptomic studies in FN are limited but could provide a valuable insight into the molecular mechanisms underlaying disease development and progression. In FD, current transcriptomic approaches have primarily been used to evaluate response to ERT [12,13]. These studies demonstrated the dynamic regulation of immune-/inflammation-related pathways and growth factor-related pathways, including innate/adaptive immune responses, lymphocyte and leukocyte proliferation, and the downregulation of genes related to oxidative phosphorylation under ERT [12]. Additionally, the transcriptomic analysis of kidney biopsies from patients with classical FN revealed that early ERT can partially restore gene expression patterns to resemble those of control subjects; however, persistent dysregulation was noted in glomerular and arterial compartments, suggesting the presence of ERT-resistant genes that may serve as biomarkers of disease progression or treatment efficacy [13]. Furthermore, transcriptomic profiling in *GLA*-edited podocyte cell lines identified 247 DEGs (136 downregulated, 111 upregulated) involved in pathways associated with oxidative stress, inflammation, fatty acid metabolism, collagen, and extracellular matrix homeostasis, kidney injury, apoptosis, autophagy and cellular stress response, providing valuable insights into the molecular basis of podocyte dysfunction in FN [14].

While transcriptomic studies have yielded valuable information on treatment response and molecular dysregulation in FD, no studies to date have specifically explored the link between gene expression profiles and the development or progression of FN, leaving a significant gap in current knowledge.

## 2. Methods

### 2.1. Study Population

The preliminary study included six Fabry patients with progressive nephropathy, enrolled at the Slovenian National Centre for Fabry Disease, General Hospital Slovenj Gradec, along with six age- and sex-matched control subjects. Progressive nephropathy was defined as an estimated glomerular filtration rate (eGFR) decline of more than 3 mL/min/1.73 m^2^ per year [15]. The inclusion and exclusion criteria for Fabry patients and control subjects have previously been established in our research [16]. eGFR was calculated using the Chronic Kidney Disease Epidemiology Collaboration (CKD-EPI) 2009 equation [17]. The study protocol was approved by the National Medical Ethics Committee of the Republic of Slovenia (0120-260/2020/6, 0120-260/2020/12, 0120-260/2020-2711-21) and written informed consent was obtained from all participants prior to enrelment in the study.

### 2.2. Sample Collection and RNA Sequencing

Peripheral blood samples were collected in Tempus™ Blood RNA Tubes (Applied Biosystems, Waltham, MA, USA), mixed vigorously, and stored at −80 °C until RNA isolation. Subsequently, total RNA was extracted using the MagMAX™ for Stabilized Blood Tubes RNA Isolation Kit (Invitrogen, Waltham, MA, USA) and manufacturer’s instructions. The concentration and integrity of the isolated RNA samples were then assessed on a NanoDrop One spectrophotometer (Thermo Fisher Scientific, Waltham, MA, USA) and an Agilent 2100 Bioanalyzer (Agilent Technologies, Santa Clara, CA, USA) with the Agilent RNA 6000 Pico Kit (Agilent Technologies, Santa Clara, CA, USA). Finally, we stored the extracted RNA samples at −80 °C until further processing. Libraries were prepared using the manufacturer’s recommended protocol, namely Illumina Stranded Total RNA Prep Ligation with Ribo-Zero Plus protocol (Illumina, San Diego, CA, USA) and Illumina RNA UD Indexes (Illumina, San Diego, CA, USA). Subsequently, paired-end sequencing with 20 million reads per sample was conducted with the Illumina NextSeq 2000 platform (Illumina, San Diego, CA, USA).

### 2.3. Bioinformatic and Statistical Analysis

First, raw sequencing data were processed using the DRAGEN RNA Pipeline (Illumina, San Diego, CA, USA) for read alignment on the human reference genome (GRCh38) and quality control. Next, the aligned reads were analyzed in R (R Foundation for Statistical Computing, Vienna, Austria). Gene-level count matrices were generated using featureCounts [18]. Differential expression analysis was then conducted using DESeq2 [19]. To account for multiple tests, *p*-values were adjusted using the false discovery rate (FDR). Finally, functional enrichment analysis was performed using Enrichr [20] to identify the significantly enriched biological pathways and processes.

Descriptive statistics were used to summarize the study population characteristics. Continuous variables were reported as median and interquartile range, while categorical variables were presented as counts and percentages. Group comparisons were performed using the two-tailed Mann–Whitney U test for continuous variables and the two-tailed Fisher’s exact test for categorical variables.

## 3. Results

### 3.1. Study Population Characteristics

The study cohort comprised six Fabry patients with progressive nephropathy and six age- and sex-matched control subjects. All Fabry patients were genetically confirmed and had classical FD phenotype. The median eGFR slope in Fabry patients with progressive nephropathy was −4.57 (−6.33 to −3.98) mL/min/1.73 m^2^ per year and the median daily proteinuria was 0.2 (0.1–1.2) g/day. The median α-Gal A enzyme activity was 5.8 (2.1–31.6)% of normal activity. All except one Fabry patient were undergoing ERT, with a median treatment duration of 11.0 (9.3–12.8) years. Additional cohort characteristics are summarized in Table 1, while further details on Fabry patient characteristics, including *GLA* variants, are provided in Table S1.

### 3.2. Differentially Expressed Genes

**Figure 1:**
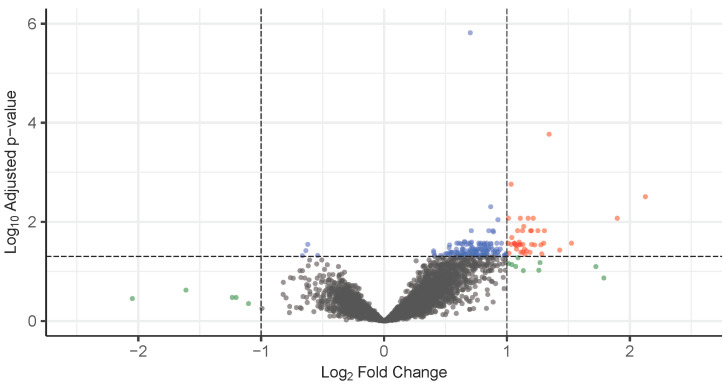
Volcano plot showing the results of the differential gene expression analysis between Fabry patients with progressive nephropathy and control subjects. A total of 41 significantly differentially expressed genes (DEGs) that met the criteria of |log_2_ fold change| > 1 and adjusted *p*-value < 0.05 are shown in red. Genes with adjusted *p*-value > 0.05 and |log_2_ fold change| < 1 are shown in gray. Genes with adjusted *p*-value < 0.05 but not meeting the fold-change criterion are shown in blue, while genes with |log_2_ fold change| > 1 but not statistically significant are marked in green.

A total of 41 DEGs were identified in Fabry patients with progressive nephropathy compared to control subjects. The threshold for significance was set at a log_2_ fold change (log2FC) > 1 and an adjusted *p*-value (FDR) < 0.05. All DEGs were upregulated in Fabry patients. The distribution of differentially expressed genes is visualized in the volcano plot (Figure 1). A comprehensive list of all DEGs, accompanied by the relevant statistical metrics, is provided in Table S2.

**Figure 2:**
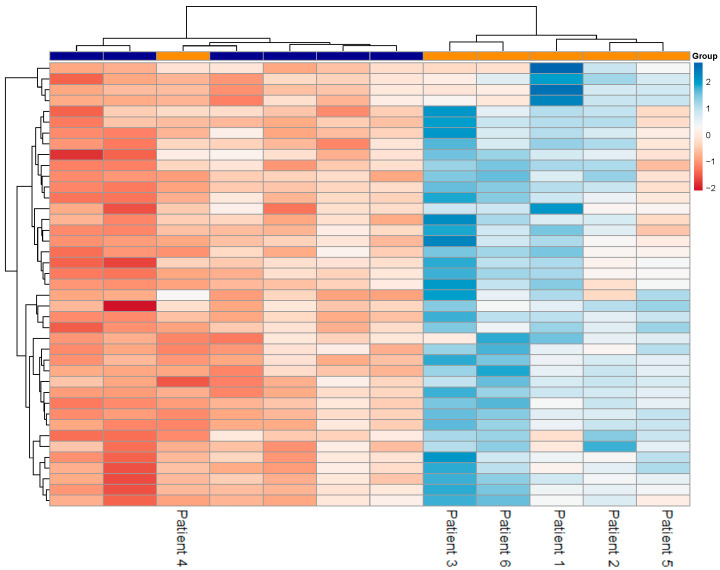
The heatmap of the 41 differentially expressed genes (DEGs) identified between Fabry patients with progressive nephropathy and control subjects. Each column represents an individual sample, and each row corresponds to DEG. Expression values are scaled across genes. Hierarchical clustering was performed using Euclidean distance and Ward’s method. The top annotation bar indicates a group: orange for Fabry patients with progressive nephropathy and blue for control subjects. One Fabry patient clusters more closely with control subjects than with the other five patients.

**Figure 3:**
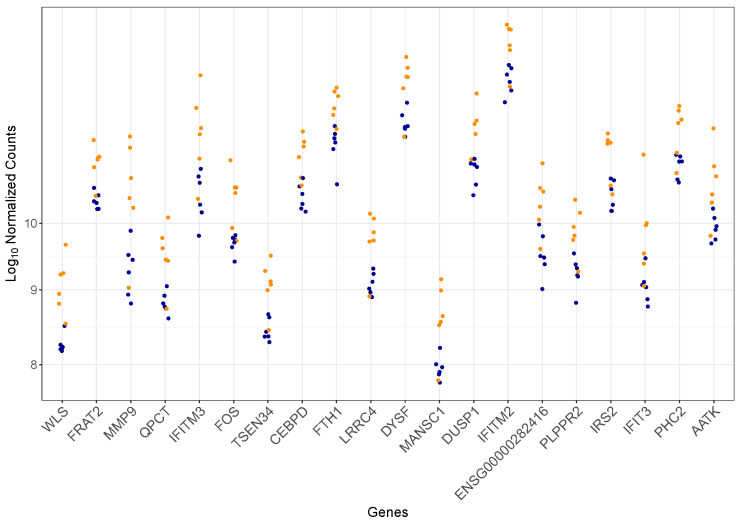
Expression levels of the top 20 differentially expressed genes (DEGs), ranked by adjusted *p*-value. Orange points represent Fabry patients with progressive nephropathy and dark blue points represent control subjects.

A heatmap illustrating the expression patterns of all 41 DEGs is presented in Figure 2. Hierarchical clustering based on gene expression differentiates Fabry patients with progressive nephropathy from control subjects delineating two discrete groups. However, one Fabry patient clustered with the control group. Furthermore, Figure 3 illustrates the expression levels of the top 20 DEGs ranked by adjusted *p*-value. The expression levels of these genes were consistently higher in Fabry patients with progressive nephropathy compared to control subjects.

### 3.3. Enrichment Analysis

**Figure 4:**
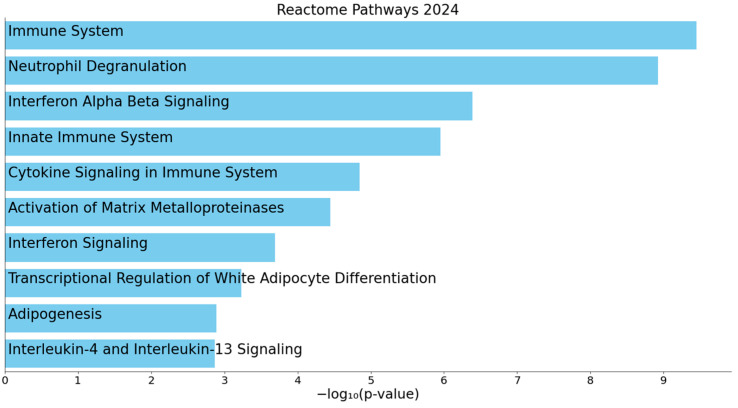
Bar plot showing the top 10 significantly enriched pathways among the differentially expressed genes (DEGs). Pathway enrichment analysis was performed using Enrichr with the Reactome Pathways 2024 gene set library [20]. Pathways are ranked by −log10 (*p*-value).

Pathway enrichment analysis of DEGs revealed the significant enrichment of immune system-related pathways, followed by neutrophil degranulation, interferon α/β signaling, innate immune system, and cytokine signaling (all *p*_adj_ < 0.001). These represent the top five pathways based on statistical significance, with the top ten pathways shown in Figure 4 and detailed in Table S3.

**Figure 5:**
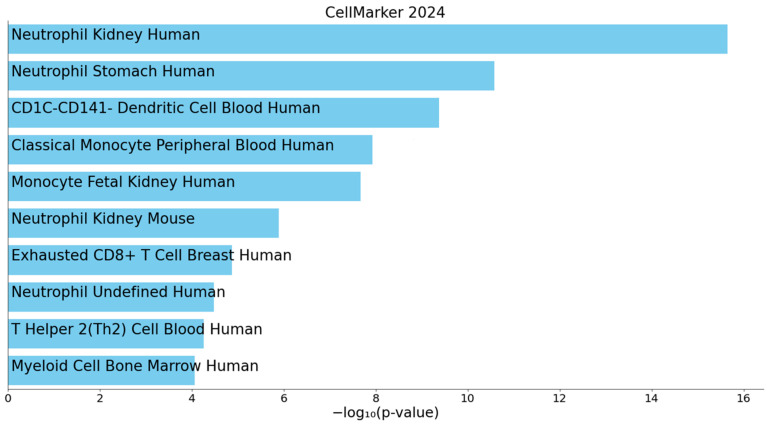
Bar plot showing the top 10 significantly enriched cell types among the differentially expressed genes (DEGs). Cell type enrichment analysis was performed using Enrichr with the CellMarker 2024 gene set library [20]. Cell types are ranked by −log10 (*p*-value).

Furthermore, cell-type enrichment analysis showed that DEGs were the most enriched in the kidney neutrophils, followed by the stomach neutrophils, CD1c- CD141- dendritic cells and classical monocytes in peripheral blood and monocytes in the fetal kidney (all *p*_adj_ < 0.001). These represent the top five enriched cell types, with the top ten shown in Figure 5 detailed in Table S4. These findings underscore the potential involvement of immune and inflammatory processes in the progression of nephropathy in FD.

## 4. Discussion

Transcriptomic studies in CKD proved to enable the characterization of altered mRNA profiles [21], thereby linking transcriptomic factors with functional molecules within cells. They provide valuable insight into disease mechanisms and facilitate the identification of potential therapeutic targets. In this proof-of-concept study focusing on factors underlying the progression of FN, we explored transcriptomic alterations in the peripheral blood samples of Fabry patients with progressive nephropathy using RNA sequencing. Our analysis identified 41 DEGs between Fabry patients and control subjects, suggesting that systemic transcriptional changes can be detected in the blood and may reflect ongoing pathophysiological processes related to FN. The DEGs exhibited an elevated expression level in Fabry patients compared to the control subjects. The most statistically significant transcriptional differences were observed for *WLS*, *FRAT2*, *MMP9*, *QPCT*, and *IFITM3*.

*WLS* and *FRAT2* are both involved in signal transduction, particularly within the Wnt signaling pathway, which plays a crucial role in cell differentiation, proliferation and fibrosis [22]. This pathway has previously been shown to be upregulated in fibrotic kidneys and is known to promote kidney interstitial fibrosis [23]. Moreover, the activation of Wnt/β-catenin signaling has been implicated in podocyte injury and the development of proteinuria [24], two key pathological features in various forms of CKD. While the role of this pathway in FN has not yet been directly studied, its involvement in DKD has been documented [25,26], suggesting a potential shared fibrotic mechanism that may also contribute to Fabry-related kidney damage.

In addition to *MMP9*, among the DEGs, upregulated *MMP25* was also identified. Together with *COL18A1*, these genes were found to be enriched in the pathway associated with the activation of matrix metalloproteinases (MMPs). MMPs are enzymes that play a pivotal role in the degradation and remodeling of the extracellular matrix (ECM) and have been identified as critical regulators of various biological processes, including inflammation, epithelial–mesenchymal transition (EMT), cell proliferation, angiogenesis, and apoptosis [27]. The dysregulation of MMPs has been implicated in various kidney diseases [28,29]. In DKD, the expression of MMPs is, at least in part, regulated by transforming growth factor β (TGF-β), which subsequently promotes extracellular matrix production, fibrosis, and podocyte injury [30]. While the specific role of MMPs in FN remains to be elucidated, prior studies have demonstrated that globotriaosylceramide (Gb3) and globotriaosylsphingosine (lyso-Gb3) can induce EMT through the release of TGF-β from tubular cells [31]. This mechanism may contribute to the upregulation of MMPs in FN, thereby promoting progressive kidney injury and fibrosis. Given its central role in fibrosis, TGF-β is currently being investigated as a potential therapeutic target to prevent kidney fibrosis and slow the progression of CKD [32].

*QPCT* and *IFITM3* have been demonstrated to be associated with the immune system. Specifically, *IFITM3* belongs to the interferon-induced transmembrane protein family and is part of a broader cluster of upregulated immune-related genes identified in the present study, including *IFIT2*, *IFIT3,* and *IFI6*. These genes were found to be significantly enriched in the interferon signaling pathway, particularly within the interferon α/β signaling pathway. While type I interferons (IFN-α and IFN-β) have traditionally been associated with viral inflammation in the kidney [33], their role in non-viral kidney inflammation remains largely unexplored, with most available data focusing on lupus nephritis [34].

When analyzing the enrichment of pathways, the top enriched pathway was the immune system, with several other immune-related pathways also being enriched, though to a lesser extent. One such pathway was the innate immune system, which plays a central role in early immune defense. The activation of the innate immune system in FD is typically initiated by signaling from dendritic cells [35]. One of the enriched immune cell types identified in our cell type enrichment analysis was also dendritic cells, specifically CD1c- CD141-dendritic cells. Interestingly, in DKD, an immune-mediated mechanism has been described where elevated glucose levels trigger Toll-like receptor 4 (TLR4) activation, leading to NF-κB activation and kidney fibrosis [36]. It is hypothesized that a similar mechanism may be involved in FN [37]. In this context, TLR4, which is expressed on both intrinsic kidney cells and infiltrating immune cells in the glomeruli and interstitial tissue [38], may be chronically activated by the persistent presence of Gb3 and lyso-Gb3 [37]. This activation triggers the release of cytokines and chemokines from immune cells, leading to the recruitment of leukocyte to the kidney, resulting in interstitial inflammation and fibrosis [39]. Supporting this, the most significantly enriched cell type in our analysis was kidney-specific neutrophils, suggesting their potential involvement in kidney inflammation. Another enriched immune-related biological process was neutrophil degranulation, which has been shown to amplify local inflammation and promote the recruitment of additional neutrophils to the site of injury, thereby contributing to the development of chronic kidney inflammation [40]. Over time, these processes contribute to tissue remodeling and fibrosis, both of which are hallmarks of FN progression [41].

Even though this is a proof-of-concept study, a limitation of this study is the relatively small sample size, as the analysis was based on six Fabry patients with progressive nephropathy and six control subjects. Consequently, the findings should be interpreted with caution and regarded as exploratory. Additionally, the further validation of the identified DEGs on an independent cohort and/or using complementary techniques such as quantitative PCR is warranted. These validation steps will be crucial to confirm the reproducibility and biological relevance of our findings and to assess their potential clinical utility. Another important limitation is that, in this preliminary study, we did not account for sex-specific differences in gene expression. This is particularly relevant in FD, where female patients often exhibit a more heterogeneous and variable clinical presentation compared to males, due to random X-chromosome inactivation [42]. Notably, one of the Fabry patients in our cohort (Patient 4, see Table S1) exhibited a gene expression profile more closely resembling that of the control group than the other Fabry patients. This finding may be indicative of a milder course of progressive nephropathy or a slower rate of disease progression. It might therefore be important that future larger studies also consider differentiating between Fabry patients with different rates of disease progression. In addition, future studies should consider ERT-related variables. In our study, although all treated patients received ERT, there was heterogeneity in both the type and duration of treatment, which may have contributed to transcriptomic variability. Notably, one untreated patient was included in our cohort and clustered with the treated Fabry patients in the heatmap (Figure 2), suggesting that, while ERT may influence gene expression, certain disease-associated transcriptional signatures may still be preserved. Due to the limited sample size and retrospective nature of the data, we were not able to perform stratified analyses based on ERT type/duration and sex in this study.

One of the most important questions in Fabry nephropathy remains whether the primary pathological events in Fabry nephropathy originate from kidney cells or from circulating immune cells. The accumulation of Gb3 has been documented in all kidney cell types of the kidney [43]; however, podocytes appear to be among the first cells affected in the early stages of Fabry nephropathy [44]. At the same time, immune cells, particularly dendritic cells and monocytes, show features of early involvement and exhibit basal proinflammatory cytokine production, which is further enhanced by inflammatory stimulation [35]. However, our data do not allow us to determine whether immune activation precedes or follows kidney injury. Clarifying the sequence of these events is critical to understanding disease mechanisms and developing therapeutic strategies. Future studies using single-cell RNA sequencing could address this limitation by identifying the earliest molecular changes in specific cell types.

Subsequent studies examining publicly available single-cell RNA sequencing datasets may provide further insight into the cellular origins of the observed transcriptomic changes. Comparison of our DEGs with existing cell type-specific expression profiles, particularly from kidney tissue and immune cell populations, would allow researchers to better determine whether the observed systemic transcriptomic signals originate primarily from kidney compartments or circulating immune cells. This integrative approach could improve the interpretation of bulk RNA sequencing data and justify more targeted experimental validation in future studies.

Despite these limitations, the findings of this study provide important preliminary insights that urge further exploration. The present study needs to be expanded into a more extensive analysis including a more comprehensive cohort of Fabry patients—not only those with progressive nephropathy, but also those with stable kidney function. This would facilitate an examination of transcriptomic differences across the entire spectrum of kidney involvement in FD. To achieve this, longitudinal assessments of gene expression might be beneficial in order to monitor molecular changes over time. This approach would enable the identification of early transcriptomic biomarkers that can predict the progression of nephropathy, validate initial findings, and provide a more comprehensive understanding of the pathophysiological mechanisms underlying FN.

## 5. Conclusions

This proof-of-concept study demonstrates that the transcriptomic profiling of peripheral blood can reveal systemic gene expression changes associated with the progression of FN. The identification of DEGs and enriched pathways related to fibrosis, inflammation, and immune activation underscores the potential of transcriptomic approaches to provide new insights into the molecular mechanisms underlying Fabry-related kidney damage. Blood sampling is minimally invasive and easily accessible but only provides an indirect picture of kidney pathology. Kidney biopsy-based transcriptomic analysis may provide further insight into local molecular changes in the affected kidney compartments. Furthermore, single-cell RNA sequencing should be considered in future studies, as it enables the analysis of gene expression at the level of individual cells. This high-resolution approach would facilitate the identification of cell type-specific transcriptional changes and improve the delineation of the cellular landscape involved in FN. Most importantly, it would advance the path towards the ultimate goal of discovering early biomarkers of disease development and progression, and identifying novel therapeutic targets to improve clinical outcomes in patients with FN.

## Figures and Tables

**Table 1:** Study cohort characteristics.

	FD-PN (*n* = 6)	Control Group (*n* = 6)	*p*-Value
Male (%)	3 (50)	3 (50)	>0.999
Age (years)	56.2 (47.5–59.4)	56.6 (48.4–60.1)	0.818
eGFR (mL/min/1.73 m^2^)	58.0 (47.0–87.8)	94.5 (93.3–96.5)	0.199
S-Cr (μmol/L)	109.0 (78.8–141.5)	69.5 (62.5–78.0)	0.078
UPCR (g/mol)	9.2 (8.7–52.8)	7.2 (6.5–7.3)	0.052
UACR (g/mol)	1.1 (1.0–508.6)	0.6 (0.4–0.8)	0.092

For continuous variables, values are presented as the median (interquartile range) and for categorical variables, values are presented as counts (percentages). eGFR, estimated glomerular filtration rate; FD-PN, Fabry patients with progressive nephropathy; S-Cr, serum creatinine; UPCR, urinary protein-to-creatinine ratio; UACR, urinary albumin-to-creatinine ratio.

## Data Availability

The data generated and analyzed in this study are available from the corresponding author upon reasonable request.

## Supplementary Materials

The following supporting information can be downloaded at https://www.mdpi.com/article/10.3390/genes16050601/s1, Table S1: Additional cohort characteristics; Table S2: Differentially expressed genes (DEGs) between Fabry patients with progressive nephropathy and control subjects; Table S3: Top 10 significantly enriched pathways among the differentially expressed genes (DEGs); Table S4: Top 10 significantly enriched cell types among the differentially expressed genes (DEGs). Reference [45] is cited in the supplementary materials.

## Abbreviations

The following abbreviations are used in this manuscript:

CKD	chronic kidney disease
DEGs	differentially expressed genes
DKD	diabetic kidney disease
ECM	extracellular matrix
eGFR	estimated glomerular filtration rate
EMT	epithelial–mesenchymal transition
ERT	enzyme replacement therapy
FD	Fabry disease
FDR	false discovery rate
FN	Fabry nephropathy
Gb3	globotriaosylceramide
Lyso-Gb3	globotriaosylsphingosine
MMPs	matrix metalloproteinases
TGF-β	transforming growth factor β
TLR4	Toll-like receptor 4
