# Supplementary material for: Transcriptomic Approach in Understanding Fabry Nephropathy: A Review of the Literature and Proof-of-Concept
Source: Genes (Basel). 2025 May 19;16(5):601. doi: 10.3390/genes16050601 (PMC12110829; doi:10.3390/genes16050601)
Supplement: Supplementary file 1 [file genes-16-00601-s001.zip › genes-3618633-supplementary.pdf]

Supplementary materials

Table S1. Additional cohort characteristics. Genetic variants and clinical phenotypes of the Fabry patients with progressive nephropathy included in the study are presented. HCMP was assessed using echocardiography and/or cardiac MRI and defined according to the literature [45]. Stroke was confirmed by appropriate image testing. Characteristic FD symptoms include Fabry-associated neuropathic pain, angiokeratoma and/or cornea verticillata.

Patient	Variant in *GLA* gene (NM_000169.2)	HCMP	Stroke	Characteristic FD symptoms	ERT
**1**	c.1088G>C, p.Arg363Pro	yes	no	yes	agalsidase alpha
**2**	c.540G>C, p.Leu180Phe	yes	yes	yes	agalsidase beta
**3**	c.1072_1074delGAG, p.358delGlu	yes	yes	yes	agalsidase alpha
**4**	c.679C>T, p.Arg227Ter	no	no	yes	agalsidase alpha, then agalsidase beta
**5**	c.815A>G, p.Asn272Ser	no	no	yes	no therapy
**6**	c.1025G>A, p.Arg342Gln	yes	no	yes	agalsidase beta, then pegunigalsidase alpha

ERT, enzyme replacement therapy; FD, Fabry disease; HCMP, hypertrophic cardiomyopathy.

Table S2. Differentially expressed genes (DEGs) between Fabry patients with progressive nephropathy and control subjects. The table lists all 41 DEGs identified using DESeq2. For each gene, the base mean expression across all samples, log_2 fold change (log_2FC), standard error (SE) of the log_2FC and the adjusted p-value (p_{adj}-value) are provided.

Gene	Base mean	log_2FC	SE(log_2FC)	p_{adj}-value
MMP25	5251	1.13	0.31	0.029
DGAT2	851	1.10	0.30	0.029
MMP9	1408	2.13	0.45	0.003
CRISPLD2	2024	1.16	0.34	0.039
PLPPR2	833	1.04	0.26	0.021
P2RX1	624	1.08	0.31	0.036
MANSC1	349	1.13	0.28	0.015
QPCT	644	1.17	0.27	0.008
WLS	461	1.34	0.25	< 0.001
IFIT3	899	1.53	0.41	0.027
IFIT2	1282	1.11	0.33	0.041
DUSP1	2678	1.19	0.29	0.015
IFI6	420	1.43	0.41	0.037
LRRC4	756	1.09	0.26	0.015
HELZ2	2693	1.02	0.31	0.043
PHC2	2738	1.06	0.28	0.027
DYSF	5033	1.25	0.30	0.015
IFITM3	2799	1.90	0.43	0.008
FCGR3B	12415	1.15	0.35	0.045
CXCR1	4605	1.11	0.31	0.029
FTH1	3669	1.14	0.27	0.012
FOS	1189	1.21	0.28	0.008
TSEN34	471	1.01	0.23	0.008
LRG1	644	1.19	0.35	0.041
NLRP6	606	1.28	0.35	0.029
FRAT2	1777	1.03	0.21	0.002
AATK	1492	1.30	0.35	0.027
ZNF467	1259	1.01	0.26	0.027
COL18A1	1004	1.14	0.33	0.036

KREMEN1	449	1.28	0.39	0.045
IFITM2	8713	1.20	0.29	0.015
IRS2	1971	1.10	0.28	0.026
FCAR	485	1.06	0.28	0.027
LILRA5	1034	1.03	0.28	0.029
PEAK3	512	1.07	0.29	0.029
NTNG2	416	1.13	0.34	0.041
MSRB1	2859	1.20	0.33	0.029
CEBPD	1929	1.11	0.25	0.008
MGAM	5585	1.23	0.34	0.029
**ENSG00000268903**	1397	1.08	0.30	0.031
**ENSG00000282416**	1137	1.31	0.32	0.015

Table S3. Top 10 significantly enriched pathways among the differentially expressed genes (DEGs). For each pathway, the adjusted p-value (p_{adj}-value) and the list of overlapping genes from the DEGs are provided. Pathway enrichment analysis was performed using Enrichr with the Reactome Pathways 2024 gene set library.

Pathway	p_{adj}-value	Overlap genes
Immune system	< 0.001	*IFITM3, MGAM, IFITM2, CEBPD, IFI6, IRS2, FOS, MMP9, FCAR, IFIT3, IFIT2, LILRA5, MMP25, LRG1, FCGR3B, CXCR1, CRISPLD2, QPCT, P2RX1, FTH1*
Neutrophil degranulation	< 0.001	*MGAM, MMP25, LRG1, FCGR3B, CXCR1, CRISPLD2, QPCT, P2RX1, FTH1, MMP9, FCAR*
Interferon alpha/beta signaling	< 0.001	*IFITM3, IFITM2, IFI6, IFIT3, IFIT2*
Innate immune system	< 0.001	*MGAM, MMP25, LRG1, FCGR3B, CXCR1, CRISPLD2, QPCT, P2RX1, FTH1, FOS, MMP9, FCAR*
Cytokine signaling in immune system	< 0.001	*IFITM3, IFITM2, CEBPD, IFI6, IRS2, FOS, MMP9, IFIT3, IFIT2*
Activation of matrix metalloproteinases	0.001	*COL18A1, MMP25, MMP9*
Interferon signaling	0.005	*IFITM3, IFITM2, IFI6, IFIT3, IFIT2*
Transcriptional regulation of white adipocyte differentiation	0.014	*HELZ2, CEBPD, ZNF467*
Adipogenesis	0.025	*HELZ2, CEBPD, ZNF467*
Interleukin-4 and interleukin-13 signaling	0.025	*CEBPD, FOS, MMP9*

Table S4. Top 10 significantly enriched cell types among the differentially expressed genes (DEGs). For each cell type, the adjusted p-value (p_{adj}-value) and the list of overlapping genes from the DEGs are provided. Cell type enrichment analysis was performed using Enrichr with the CellMarker 2024 gene set library.

Pathway	p_{adj}-value	Overlap genes
Neutrophil kidney human	< 0.001	*DGAT2, LRG1, IFITM2, FCGR3B, CXCR1, QPCT, FOS, MMP9, FCAR, MSRB1*
Neutrophil stomach human	< 0.001	*MGAM, FCGR3B, CXCR1, CRISPLD2, DYSF, FCAR*
CD1c- CD141- dendritic cell blood human	< 0.001	*IFITM3, IFITM2, FCGR3B, DUSP1, P2RX1, FTH1, IFI6, FCAR, IFIT3, IFIT2*
Classical monocyte peripheral blood human	< 0.001	*IFITM3, IFI6, FCAR, IFIT3, IFIT2*
Monocyte fetal kidney human	< 0.001	*MGAM, DGAT2, LRG1, IFITM2, FCGR3B, CEBPD, DUSP1, CRISPLD2, QPCT, P2RX1, FCAR, MSRB1*
Neutrophil kidney mouse	< 0.001	*LRG1, DUSP1, FOS, MMP9, MSRB1*
Exhausted CD8+ T cell breast human	< 0.001	*IFITM3, IFI6, IFIT3*
Neutrophil undefined human	< 0.001	*FCGR3B, CXCR1, FCAR*
T helper 2 (Th2) cell blood human	< 0.001	*IFIT3, IFIT2*
Myeloid cell bone marrow human	0.001	*FCGR3B, CRISPLD2, DYSF, FCAR*
